# Supplementary material for: SiMYB19 from Foxtail Millet (Setaria italica) Confers Transgenic Rice Tolerance to High Salt Stress in the Field
Source: Int J Mol Sci. 2022 Jan 11;23(2):756. doi: 10.3390/ijms23020756 (PMC8775554; doi:10.3390/ijms23020756)
Supplement: Supplementary file 1 [file ijms-23-00756-s001.zip › ijms-1525110-supplementary.pdf]

Supplementary Materials:

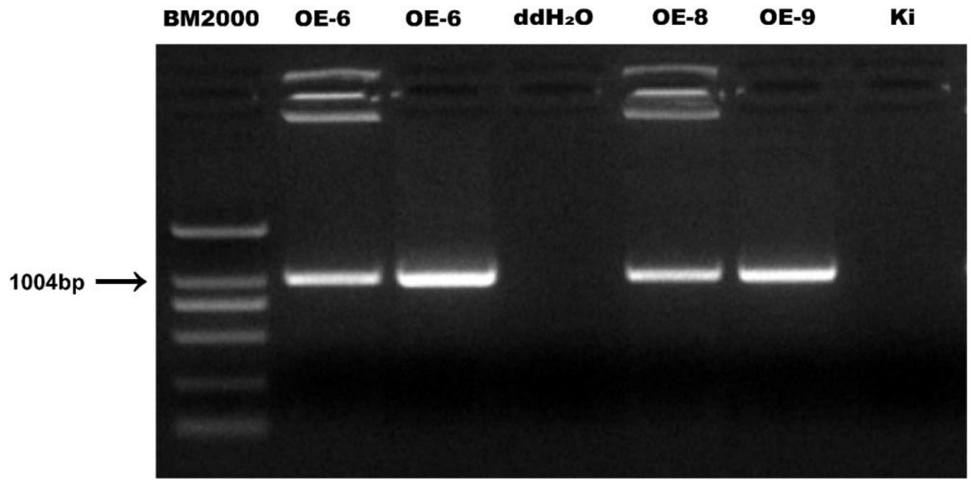

Figure S1. PCR analysis of T3 generation *SiMYB19* transgenic rice plants. BM2000, DNA Marker; Ki, Kitaake; OE-6, OE-8, and OE-9, transgenic rice lines; ddH2O, negative control.

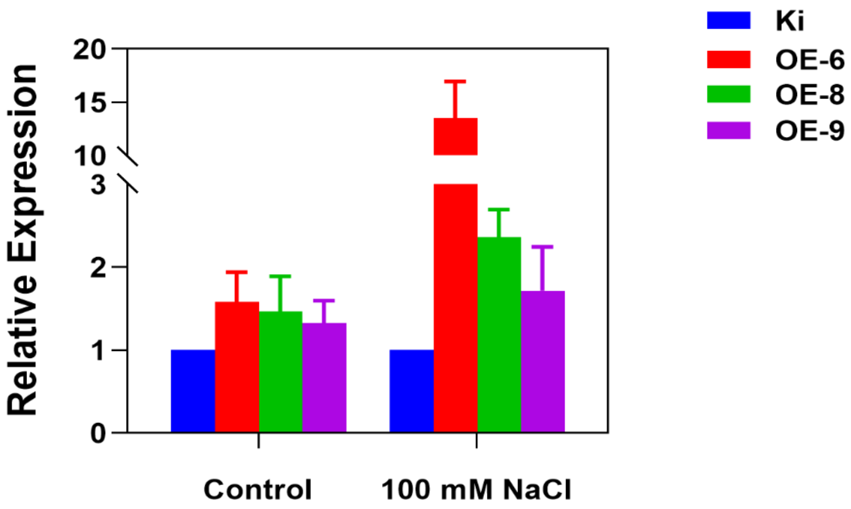

Figure S2. Relative *SiMYB19* expression in transgenic rice and WT under normal and salt stress conditions. Ki, Kitaake; OE-6, OE-8, and OE-9, transgenic rice lines; Data are means ± SD of three independent experiments.

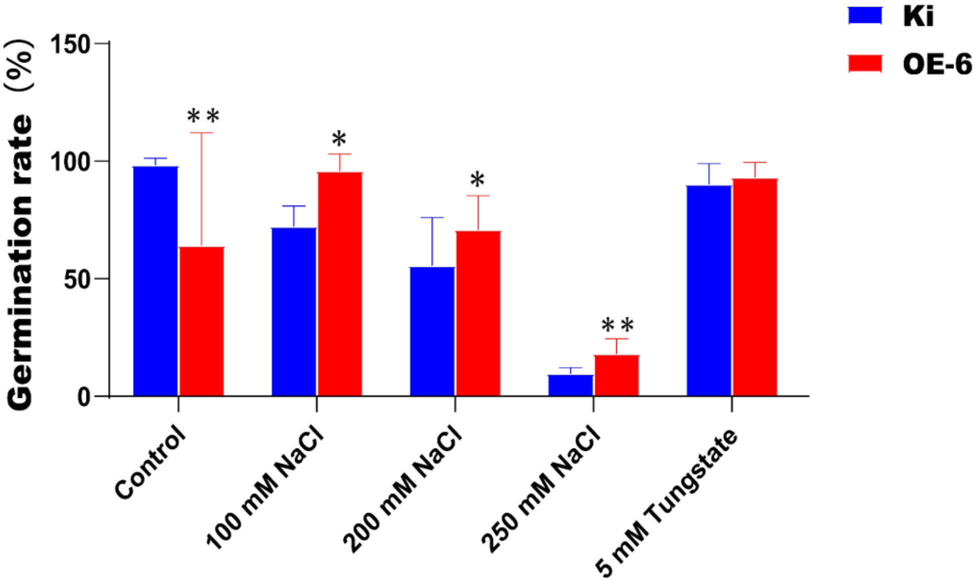

Figure S3. Final germination rates of WT and transgenic rice OE-6 subjected to various salt concentrations. The germination rates of treatment after 8d. Data are means ±SD (n = 3), ***P* < 0.01, **P* < 0.05, t-test.

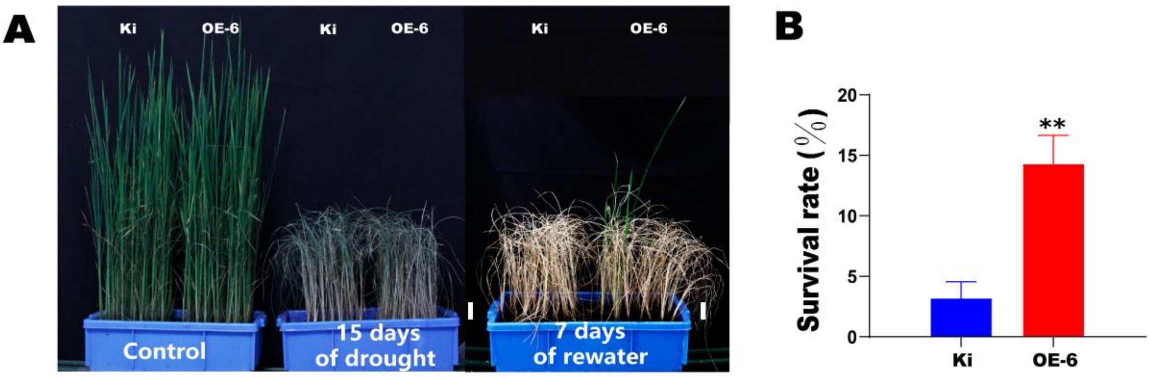

Figure S4. Drought stress tolerance analysis of transgenic line OE-6 and WT in soil. (A) Growth of transgenic OE-6 and WT seedlings under control and drought conditions in soil. Phenotypes of transgenic line and WT after 15 d drought and 7 d recovery. Bar = 5 cm. (B) Survival rates of transgenic line OE-6 and WT after post-drought irrigation. ***P* < 0.01, *t*-test.

Table S1. Primers used for the qRT-PCR analysis of the *SiMYB19* and primers for related experiments

For gene	Primer F (5'-3')	Primer R (5'-3')	Function
SiMYB19	AGCAGAAGCCCCTCCTGTAG	GTGTCGAGCCTAGTTGAGCC	Transgenic detection
p16318GFP-SiMYB19	TATCTCTAGAGGATCCATGGGGTGCAAGGCGT	TGCTCACCATGGATCCGTTGAGCCCGAAGTACTCG	Subcellular localization
pGBKT7-SiMYB19	AGGAGGACCTGCATATGATGGGGTGCAAGGCGT	GCCTCCATGGCCATATGGTTGAGCCCGAAGTACTC	Activates transcription
RT-SiMYB19	GTTCCAGGAGCACTTCGACA	CCCTTCACCAACGATCACCA	Real-time PCR
RT-OsNCED3	CACTCCCTTCTCATTCCC	AGCCCTTGTTCAGGTTAA	Real-time PCR
RT-OsPK1	TTCAAGCTTTGCTGTTGCCG	ATGCGACATCTCATCACCCC	Real-time PCR
RT-OsABF2	GGCATGCCATCTGTGTTTGTT	GACAGCAACTTCGTCTCGCA	Real-time PCR
RT-OsLEA7	GGACAAAACTCAAGAGACAG	CATCGTGTGCCTTGTTCTTGAT	Real-time PCR
RT-OsCHS	AGTGTCATGGATGGTGAGGT	TCAACCAAACTTGAATGCACATC	Real-time PCR
RT-OsFAR2	GGTGCCTGTAGTCGGAGATG	CTGAATGGCCCTACGGTGTT	Real-time PCR
OsActin	CCTTCAACACCCCTGCTATG	CAATGCCAGGGAACATAGTG	Real-time PCR
SiActin	GGCAAACAGGGAGAAGATGA	GAGGTTGTCGGTAAGGTCACG	Real-time PCR

Table S2. Composition of rice nutrient solution

Component	Reagent name (Analytical grade)	Amount of stock solution（g/4L）	Final concentration (ppm)
Macro elements			
N	$(NH_4)_2SO_4$	603.2	40
P	$NaH_2PO_4 \cdot 2H_2O$	160.9	10
K	K_2SO_4	285.6	40
Ca	$CaCl_2 \cdot 2H_2O$	469.4	40
Mg	$MgSO_4 \cdot 7H_2O$	1296.0	40
Micro elements			
Mn	$MnCl_3 \cdot 4H_2O$	6.000	0.50
Mo	$(NH_4)_6Mo_7O_{24} \cdot 4H_2O$	0.296	0.05
Zn	$ZnSO_4 \cdot 7H_2O$	0.140	0.01
B	H_3BO_3	3.736	0.20
Cu	$CuSO_4 \cdot 5H_2O$	0.124	0.01
Fe	$FeCl_3 \cdot 6H_2O$	30.800	2.00
Citric acid	$C_6H_8O_7 \cdot H_2O$	47.600	
